# Supplementary material for: A Likelihood Approach for Real-Time Calibration of Stochastic Compartmental Epidemic Models
Source: PLoS Comput Biol. 2017 Jan 17;13(1):e1005257. doi: 10.1371/journal.pcbi.1005257 (PMC5240920; doi:10.1371/journal.pcbi.1005257)
Supplement: S1 File — (TAR.GZ) [file pcbi.1005257.s014.tar.gz › HSPH_Online-SI-Revision/IPoi09/n100-extreme/n100-extreme_BENCH_table-4.pdf]

1	R0	{2.76052, 16.3913}	{2.52186, 12.8824}	{{2.4534, 12.3139}, {2.59907, 13.5234}}	{8.6453, 21.4073}	inf	10 419.	8885.43	{2167., 17 910.}	49.3832	0.336184	newly	5804.	6061.82	{1629., 10 855.}	50.4564	0.286367	Reff	2.28524	2.1113	{1.72131, 2.44626}	11.4223	ar	92 090.	89 554.3	{88 443., 90 594.}	2.75351
2	R0	{2.18202, 15.1734}	{2.48143, 15.7026}	{{2.48143, 15.7026}, {2.48143, 15.7026}}	{13.7217, 3.48768}	inf	10 970.	19 795.7	{13 638., 22 973.}	87.4173	0.289718	newly	5914.	8750.9	{7066., 10 190.}	54.6183	0.206176	Reff	1.6841	1.10659	{0.67815, 1.84122}	40.1203	ar	84 264.	89 043.	{88 838., 89 242.}	5.67148
3	R0	{2.57891, 12.5971}	{2.73634, 13.3345}	{{2.73634, 13.3345}, {2.73634, 13.3345}}	{6.10452, 5.85373}	inf	8525.	14 917.	{4827., 23 604.}	96.0692	0.338608	newly	6571.	9350.13	{3752., 13 428.}	63.4312	0.267015	Reff	2.202	1.98452	{1.45026, 2.52739}	17.1798	ar	90 302.	91 899.1	{91 744., 92 094.}	1.76862
4	R0	{2.5856, 9.28508}	{2.80081, 9.90389}	{{2.25976, 7.03829}, {2.91001, 10.5991}}	{10.9338, 11.6228}	inf	2564.	3059.85	{477., 6845.}	82.497	0.384717	newly	3492.	3831.06	{655., 8343.}	73.8639	0.371734	Reff	2.47933	2.68861	{2.49297, 2.87541}	10.7405	ar	90 312.	92 556.2	{92 608., 93 492.}	3.18602
5	R0	{2.4406, 12.1341}	{2.76251, 13.3728}	{{2.7943, 13.5962}, {2.7943, 13.5962}}	{14.9099, 13.3239}	inf	4472.	9820.31	{2663., 19 163.}	142.264	0.412537	newly	3644.	6733.87	{2154., 11 955.}	107.096	0.361243	Reff	2.2517	2.28808	{1.77343, 2.67551}	13.4504	ar	88 597.	91 709.	{92 205., 92 620.}	4.82922
6	R0	{2.72155, 15.4094}	{2.58092, 13.7736}	{{2.58092, 13.7736}, {2.58092, 13.7736}}	{5.16728, 10.6156}	inf	8392.	16 015.2	{3810., 24 183.}	111.874	0.383091	newly	5118.	9114.84	{2767., 12 320.}	96.6818	0.347149	Reff	2.34317	1.74109	{1.15341, 2.41879}	27.2339	ar	91 682.	90 300.2	{90 086., 90 511.}	1.50716
7	R0	{2.63781, 14.3935}	{2.89334, 18.7697}	{{2.89334, 18.7697}, {2.89334, 18.7697}}	{9.6872, 30.404}	inf	9079.	9380.02	{1421., 17 396.}	54.2907	0.32632	newly	5875.	4379.85	{757., 7675.}	40.8672	0.324214	Reff	2.22919	2.44781	{2.03946, 2.82645}	13.8947	ar	91 192.	93 255.9	{93 130., 93 358.}	2.26327
8	R0	{2.82391, 16.104}	{2.58092, 13.7736}	{{2.58092, 13.7736}, {2.58092, 13.7736}}	{8.60474, 14.4709}	inf	8948.	11 210.1	{1786., 20 171.}	63.8263	0.335273	newly	5170.	6926.67	{1328., 11 434.}	66.2589	0.32158	Reff	2.41478	2.04388	{1.54984, 2.50135}	16.7582	ar	92 664.	90 300.2	{90 086., 90 511.}	2.55093
9	R0	{2.80645, 14.6457}	{2.96917, 17.9534}	{{2.8833, 17.1847}, {2.98558, 18.1089}}	{5.89199, 22.5848}	inf	5809.	5705.48	{767., 11 265.}	61.7806	0.38653	newly	4055.	3008.51	{466., 5771.}	50.7222	0.396932	Reff	2.54301	2.70025	{2.45845, 2.95047}	9.13672	ar	92 567.	93 793.7	{93 323., 94 067.}	1.35684
10	R0	{1.86213, 5.4271}	{2.01138, 6.41061}	{{1.98388, 6.25659}, {1.98388, 6.25659}}	{8.01494, 18.1223}	inf	1977.	3592.66	{195., 8514.}	120.691	0.445812	newly	4393.	5821.14	{430., 11 574.}	76.6767	0.380357	Reff	1.77612	1.8505	{1.6633, 1.97842}	7.11892	ar	75 362.	79 657.2	{78 771., 79 873.}	5.69943
11	R0	{2.27349, 14.6338}	{2.76421, 16.5539}	{{2.74988, 16.4329}, {2.74988, 16.4329}}	{21.5845, 13.1207}	inf	10 491.	17 200.5	{5436., 26 870.}	85.2737	0.317351	newly	6044.	8155.26	{3160., 11 199.}	57.0627	0.252153	Reff	1.80677	1.85566	{1.16666, 2.58164}	22.2994	ar	85 805.	92 228.8	{91 910., 92 833.}	7.48648
12	R0	{2.77261, 14.1894}	{2.75274, 17.3228}	{{2.75274, 17.3228}, {2.75274, 17.3228}}	{0.716653, 22.0827}	inf	6492.	5445.55	{800., 11 672.}	53.0112	0.391886	newly	4508.	2896.77	{478., 5932.}	48.6411	0.421801	Reff	2.48165	2.50612	{2.21521, 2.71806}	6.14303	ar	92 508.	92 081.8	{91 850., 92 257.}	0.460762
13	R0	{2.65402, 19.3935}	{2.53741, 16.1789}	{{2.53741, 16.1789}, {2.53741, 16.1789}}	{4.39371, 16.5757}	inf	11 250.	17 091.9	{7125., 23 602.}	68.4153	0.266	newly	4926.	8068.66	{3916., 10 268.}	76.5673	0.277447	Reff	2.14402	1.62338	{1.1071, 2.22967}	26.739	ar	91 184.	89 740.	{89 517., 89 990.}	1.58356
14	R0	{1.75297, 4.45354}	{2.8594, 16.8014}	{{2.73108, 15.6124}, {2.96398, 17.7348}}	{63.1172, 277.259}	inf	699.	1679.82	{305., 3950.}	167.706	0.403961	newly	1855.	1013.71	{197., 2209.}	57.525	0.497062	Reff	1.72278	2.78484	{2.66406, 2.94371}	61.6478	ar	71 263.	92 965.5	{91 920., 93 926.}	30.4542
15	R0	{1.99928, 7.1501}	{2.71225, 12.7047}	{{2.71225, 12.7047}, {2.71225, 12.7047}}	{35.6613, 77.6856}	inf	3389.	5484.	{900., 10 649.}	95.1218	0.350603	newly	4922.	4341.32	{829., 8002.}	47.4794	0.316344	Reff	1.85853	2.4656	{2.22977, 2.6717}	32.6637	ar	79 602.	91 656.5	{91 499., 91 836.}	15.1434
16	R0	{2.67398, 10.3134}	{2.95134, 15.2591}	{{2.95376, 15.278}, {2.95376, 15.278}}	{10.3724, 47.9542}	inf	4133.	8325.77	{637., 18 342.}	132.541	0.418943	newly	4823.	5108.61	{481., 10 374.}	54.7384	0.322054	Reff	2.49563	2.55212	{2.05738, 2.92546}	10.6546	ar	91 289.	93 684.3	{93 535., 93 846.}	2.62384
17	R0	{2.44634, 16.3226}	{2.75964, 18.1443}	{{2.73737, 17.9172}, {2.93978, 19.9817}}	{12.8067, 11.1606}	inf	10 530.	10 506.4	{2731., 20 522.}	55.0331	0.32906	newly	5419.	4903.48	{1340., 8999.}	46.3149	0.303317	Reff	1.9738	2.25429	{1.71387, 2.68299}	20.0044	ar	88 710.	92 111.5	{91 759., 93 547.}	3.83446
18	R0	{2.02168, 10.1676}	{2.73799, 17.9255}	{{2.73737, 17.9172}, {2.73737, 17.9172}}	{35.4315, 76.2998}	inf	4597.	6888.27	{907., 14 326.}	95.0252	0.391885	newly	4153.	3388.71	{482., 6694.}	51.2315	0.379076	Reff	1.82331	2.42018	{2.06729, 2.69776}	32.7681	ar	80 299.	91 925.2	{91 759., 92 106.}	14.4786
19	R0	{2.89969, 15.009}	{2.94151, 17.6978}	{{2.8833, 17.1847}, {2.98558, 18.1089}}	{2.46753, 17.9144}	inf	6390.	5780.45	{640., 13 370.}	65.8316	0.428426	newly	4254.	3051.1	{366., 6668.}	54.6721	0.4321	Reff	2.60468	2.66826	{2.31483, 2.95581}	8.26595	ar	93 349.	93 593.4	{92 554., 94 045.}	0.5674
20	R0	{2.68933, 7.34928}	{2.71698, 12.256}	{{2.25325, 9.0684}, {2.95209, 14.037}}	{7.02194, 67.1373}	inf	478.	534.37	{115., 1149.}	76.8585	0.366154	newly	1059.	508.685	{94., 1258.}	59.6673	0.522054	Reff	2.66728	2.70003	{2.32686, 2.9412}	6.91317	ar	91 574.	91 338.9	{86 823., 93 702.}	2.19197
21	R0	{2.15352, 9.28074}	{2.50912, 11.3499}	{{2.32678, 10.0266}, {2.71225, 12.8589}}	{16.5123, 22.2948}	inf	3961.	5886.57	{1193., 13 571.}	90.0402	0.342839	newly	4282.	5030.51	{1040., 10 964.}	66.0258	0.317605	Reff	1.99267	2.25713	{1.91257, 2.61395}	16.756	ar	83 845.	89 157.8	{86 404., 91 726.}	6.33644
22	R0	{2.21953, 9.04387}	{2.6386, 11.0897}	{{2.48617, 9.99279}, {2.79531, 12.1249}}	{18.8812, 23.0343}	inf	3707.	4870.09	{608., 10 969.}	91.353	0.427896	newly	4322.	4496.56	{622., 9881.}	70.5034	0.412068	Reff	2.06581	2.4419	{2.04684, 2.72515}	19.4574	ar	84 856.	91 039.6	{89 097., 92 505.}	7.28715
23	R0	{2.21987, 14.5347}	{2.86095, 19.9929}	{{2.86095, 19.9929}, {2.86095, 19.9929}}	{28.8792, 37.5529}	inf	10 313.	17 896.9	{7195., 26 659.}	90.9179	0.316347	newly	5905.	7013.72	{3327., 9772.}	42.3931	0.211395	Reff	1.76493	1.92004	{1.33192, 2.5318}	22.9741	ar	85 067.	93 033.	{92 897., 93 188.}	9.36434
24	R0	{2.84268, 17.9659}	{2.53445, 17.6233}	{{2.41197, 16.4137}, {2.59755, 18.2464}}	{10.8428, 3.96795}	inf	10 277.	12 271.2	{2770., 21 253.}	62.276	0.311918	newly	5220.	5570.41	{1499., 9002.}	49.2636	0.277459	Reff	2.35894	1.91482	{1.35387, 2.47261}	20.3991	ar	92 836.	89 504.1	{87 904., 90 594.}	3.58901
25	R0	{2.52706, 10.3679}	{2.73737, 17.9172}	{{2.73737, 17.9172}, {2.73737, 17.9172}}	{8.32232, 72.8142}	inf	4409.	4039.34	{489., 8967.}	62.9361	0.421882	newly	4655.	2081.03	{259., 4472.}	58.8829	0.571467	Reff	2.33579	2.5563	{2.3364, 2.71629}	10.4308	ar	89 623.	91 915.1	{91 759., 92 090.}	2.55749
26	R0	{2.62835, 19.1088}	{2.87827, 19.2093}	{{2.73737, 17.9172}, {2.93978, 19.9817}}	{9.50845, 2.85454}	inf	12 549.	17 678.1	{6020., 27 242.}	67.1505	0.260887	newly	5505.	7216.6	{2852., 10 214.}	54.4487	0.223173	Reff	2.05547	1.92528	{1.19722, 2.60994}	23.6203	ar	90 908.	93 097.5	{92 147., 93 674.}	2.40843
27	R0	{2.76831, 19.4155}	{2.76971, 15.9605}	{{2.58655, 14.3523}, {2.80057, 16.2348}}	{1.97713, 17.7951}	inf	16 382.	23 182.5	{18 074., 27 413.}	53.3329	0.216232	newly	6900.	10 401.5	{8776., 11 799.}	59.9213	0.22467	Reff	1.96727	1.30144	{0.714593, 1.8481}	38.4776	ar	92 175.	92 145.6	{90 209., 92 639.}	0.660537
28	R0	{2.96609, 16.8903}	{1.81768, 5.68021}	{{1.81768, 5.68021}, {1.81768, 5.68021}}	{38.718, 66.37}	inf	11 248.	9449.92	{5681., 11 905.}	19.2516	0.142152	newly	6075.	12 351.7	{9668., 14 686.}	110.262	0.33796	Reff	2.44352	1.22696	{0.836296, 1.56831}	49.7875	ar	94 024.	73 876.1	{73 322., 74 338.}	21.4285
29	R0	{2.17046, 14.39}	{2.7811, 19.6712}	{{2.7811, 19.6712}, {2.7811, 19.6712}}	{28.1341, 36.7005}	inf	8850.	11 379.6	{2541., 18 694.}	64.0147	0.305908	newly	5160.	4816.07	{1215., 7693.}	40.8469	0.272014	Reff	1.78813	2.23236	{1.80071, 2.65217}	27.4913	ar	84 001.	92 343.4	{92 163., 92 489.}	9.93129
30	R0	{2.88772, 12.4647}	{1.97406, 5.41891}	{{1.71582, 3.97874}, {2.27423, 6.876}}	{31.6394, 56.5259}	inf	3959.	7115.	{1362., 12 807.}	109.742	0.362883	newly	3670.	12 026.3	{3341., 17 947.}	236.387	0.496029	Reff	2.71599	1.62379	{1.2555, 1.94331}	40.2137	ar	93 273.	77 999.6	{69 607., 81 013.}	16.375
31	R0	{2.46831, 8.14308}	{2.14862, 5.82419}	{{1.88643, 4.56746}, {2.55331, 7.74418}}	{16.3835, 29.7776}	inf	1290.	1877.83	{295., 3516.}	89.8915	0.352552	newly	2181.	4251.07	{611., 8498.}	126.708	0.379786	Reff	2.41433	2.11626	{1.75653, 2.61439}	16.2973	ar	88 762.	82 367.6	{75 886., 91 504.}	8.81
32	R0	{1.93236, 7.38987}	{1.83672, 5.79293}	{{1.83544, 5.78475}, {1.83544, 5.78475}}	{4.94949, 21.6099}	inf	2899.	4442.84	{675., 8272.}	89.3971	0.358404	newly	4137.	7262.87	{1347., 12 558.}	103.549	0.361847	Reff	1.80908	1.6355	{1.45352, 1.8069}	9.71184	ar	77 654.	74 581.6	{74 131., 74 981.}	3.95655
33	R0	{2.82251, 17.6954}	{1.79416, 6.4005}	{{1.7459, 6.01426}, {1.7459, 6.01426}}	{36.434, 63.8296}	inf	8313.	9027.15	{3053., 11 015.}	32.7	0.176344	newly	4307.	10 456.	{5140., 12 455.}	152.081	0.419803	Reff	2.44362	1.25571	{0.93465, 1.61457}	49.0049	ar	92 745.	72 600.2	{70 662., 71 917.}	21.7206
34	R0	{1.87083, 12.3994}	{2.04024, 11.5428}	{{2.04871, 11.6322}, {2.04871, 11.6322}}	{9.05523, 6.90825}	inf	6523.	11 794.2	{5677., 16 129.}	90.6157	0.281255	newly	4361.	7659.86	{4256., 9685.}	82.9282	0.264055	Reff	1.57679	1.39459	{0.971375, 1.80661}	19.006	ar	76 282.	80 841.1	{80 541., 81 192.}	5.98778
35	R0	{2.95779, 16.3003}	{2.84033, 15.4237}	{{2.86118, 15.5722}, {2.86118, 15.5722}}	{3.97137, 5.37773}	inf	6128.	9682.43	{2565., 18 448.}	86.1522	0.323473	newly	3852.	5685.92	{1792., 9977.}	74.0288	0.297104	Reff	2.67834	2.37603	{1.80809, 2.73781}	12.4205	ar	93 873.	92 744.6	{92 689., 93 174.}	1.20209
36	R0	{2.18718, 14.261}	{2.15677, 10.1363}	{{2.15677, 10.1363}, {2.15677, 10.1363}}	{1.39037, 28.9229}	inf	9128.	15 094.8	{12 105., 17 740.}	67.7864	0.22149	newly	5307.	9769.29	{7127., 12 272.}	84.6955	0.257789	Reff	1.78461	0.8604	{0.583751, 1.2674}	52.1009	ar	84 379.	83 487.1	{83 182., 83 850.}	1.05707
37	R0	{2.44912, 9.31118}	{2.64776, 10.0415}	{{2.28968, 7.89302}, {2.74056, 10.6768}}	{9.80322, 11.8036}	inf	3260.	4308.57	{952., 10 091.}	75.7953	0.342576	newly	3995.	4778.22	{1179., 10 125.}	63.6669	0.317992	Reff	2.30876	2.46538	{2.14827, 2.68109}	10.1539	ar	88 442.	90 930.7	{86 230., 91 960.}	3.45789
38	R0	{2.05949, 5.72514}	{2.64253, 11.8498}	{{2.13505, 8.31782}, {2.95209, 14.037}}	{28.3098, 106.978}	inf	809.	1455.37	{274., 3044.}	117.861	0.35325	newly	2064.	1431.75	{257., 3074.}	57.4585	0.409456	Reff	2.02491	2.54544	{2.10366, 2.89597}	25.7961	ar	81 587.	89 860.	{83 027., 93 733.}	10.1401
39	R0	{1.75505, 8.52404}	{2.6825, 16.8653}	{{2.51743, 15.191}, {2.8568, 18.4275}}	{52.8448, 97.8552}	inf	4468.	9118.29	{1983., 16 438.}	128.975	0.396318	newly	4543.	4710.65	{1148., 7799.}	46.9028	0.275774	Reff	1.55847	2.24768	{1.84543, 2.56739}	44.3471	ar	70 925.	91 215.2	{89 474., 92 996.}	28.608
40	R0	{2.24724, 13.7861}	{2.00937, 8.71836}	{{2.00488, 8.68514}, {2.00488, 8.68514}}	{10.5849, 36.7598}	inf	10 101.	13 104.4	{8487., 15 486.}	36.4158	0.137846	newly	6145.	10 871.9	{8292., 12 327.}	77.6172	0.244404	Reff	1.78404	1.19158	{0.755619, 1.53666}	33.2729	ar	85 257.	79 971.3	{79 407., 80 337.}	6.1997
41	R0	{1.77635, 8.62406}	{2.03307, 8.59564}	{{2.03307, 8.59564}, {2.03307, 8.59564}}	{14.4521, 0.329543}	inf	5330.	8508.27	{2529., 15 260.}	83.4768	0.287661	newly	5334.	8225.44	{3043., 12 776.}	73.7185	0.260653	Reff	1.53452	1.61618	{1.18016, 1.9292}	15.5189	ar	72 941.	80 504.1	{80 124., 80 869.}	10.3689
42	R0	{1.80397, 5.4932}	{2.309, 7.90141}	{{2.20704, 7.29612}, {2.60471, 9.67274}}	{27.9953, 43.8398}	inf	2276.	8525.73	{1700., 16 752.}	286.022	0.517107	newly	4341.	9940.67	{2484., 16 748.}	145.241	0.37473	Reff	1.7106	1.93947	{1.45846, 2.47838}	20.679	ar	73 232.	86 541.3	{84 283., 90 547.}	18.1741
43	R0	{2.83096, 16.7077}	{2.7811, 19.6712}	{{2.7811, 19.6712}, {2.7811, 19.6712}}	{1.76124, 17.7373}	inf	8628.	7538.17	{1427., 12 898.}	49.2296	0.342408	newly	4809.	3335.77	{683., 5553.}	43.6799	0.354955	Reff	2.43862	2.43197	{2.1264, 2.70882}	8.10747	ar	92 740.	92 343.4	{92 163., 92 489.}	0.427665
44	R0	{2.73678, 11.6021}	{2.96049, 12.7164}	{{2.93502, 12.6864}, {2.9884, 12.8326}}	{8.63663, 10.4016}	inf	6552.	13 069.5	{2296., 23 407.}	127.324	0.396479	newly	6185.	9312.53	{2128., 14 916.}	80.1737	0.316602	Reff	2.44682	2.30212	{1.73737, 2.88405}	15.6808	ar	92 003.	93 703.3	{93 493., 94 095.}	2.07693
45	R0	{2.14235, 6.98601}	{2.9695, 15.3536}	{{2.95376, 15.278}, {2.99911, 15.6207}}	{38.6093, 119.776}	inf	2850.	4335.58	{574., 10 307.}	99.22	0.370313	newly	4808.	2866.92	{460., 6542.}	56.5415	0.464875	Reff	2.02463	2.76829	{2.49465, 2.94908}	36.7308	ar	82 974.	93 817.8	{93 536., 94 085.}	13.0689
46	R0	{2.23409, 9.21289}	{2.84159, 14.5856}	{{2.48312, 11.958}, {2.91192, 15.1048}}	{27.1923, 58.3174}	inf	2544.	3683.61	{313., 9403.}	102.92	0.441049	newly	3017.	2550.59	{252., 6094.}	65.079	0.461822	Reff	2.12542	2.66802	{2.35569, 2.89611}	25.6961	ar	85 163.	92 631.	{89 154., 93 531.}	8.76902
47	R0	{2.12258, 8.15265}	{1.4804, 3.12918}	{{1.4804, 3.12918}, {1.4804, 3.12918}}	{30.2547, 61.6176}	inf	2945.	2526.39	{214., 5764.}	58.6428	0.421051	newly	3839.	7113.45	{818., 12 926.}	114.693	0.392625	Reff	1.99728	1.33434	{1.13868, 1.46727}	33.1925	ar	82 690.	57 005.2	{56 101., 57 732.}	31.0615
48	R0	{2.71771, 10.4636}	{2.55349, 10.4791}	{{2.48617, 9.99279}, {2.79531, 12.1249}}	{7.24177, 7.5333}	inf	2061.	1962.31	{207., 4618.}	69.1405	0.445739	newly	2437.	2135.09	{209., 4766.}	64.4578	0.445026	Reff	2.62862	2.48122	{2.24389, 2.76671}	7.5683	ar	91 716.	89 926.7	{88 931., 92 479.}	2.33978
49	R0	{2.44369, 6.66477}	{2.61876, 8.03792}	{{2.45236, 7.26016}, {2.77832, 8.74498}}	{8.21647, 21.9092}	inf	1213.	1755.51	{255., 3711.}	88.622	0.340392	newly	3032.	2973.79	{429., 5960.}	60.5216	0.342029	Reff	2.39345	2.55919	{2.3514, 2.7577}	7.78898	ar	88 827.	90 685.7	{88 503., 92 510.}	2.32079
50	R0	{2.77383, 17.9775}	{2.81825, 18.2716}	{{2.81891, 18.2812}, {2.81891, 18.2812}}	{1.61654, 1.70886}	inf	14 883.	12 885.8	{3267., 23 994.}	43.1987	0.287402	newly	6948.	5851.32	{1755., 9948.}	37.2881	0.255543	Reff	2.0682	2.17756	{1.53569, 2.67593}	17.2236	ar	92 334.	92 648.6	{92 506., 92 856.}	0.348105
